# Fibrillar Bundles as Fibrous Filler Materials for Attaining Cell Anisotropy in Bioprinting

**DOI:** 10.1002/adhm.202503767

**Published:** 2025-11-07

**Authors:** Sven Heilig, Zan Lamberger, Lys Sprenger, Vivien Priebe, Camilla Mussoni, Denitsa Docheva, Kristina Andelovic, Jürgen Groll, Sahar Salehi, Gregor Lang, Matthias Ryma

**Affiliations:** ^1^ Department for Functional Materials in Medicine and Dentistry University Hospital of Würzburg Pleicherwall 2 D‐97070 Würzburg Germany; ^2^ Department of Biomaterials Faculty of Engineering Science Universität Bayreuth Prof.‐Rüdiger‐Bormann. Str. 1 95447 Bayreuth Germany; ^3^ Department of Musculoskeletal Tissue Regeneration Orthopaedic Hospital König‐Ludwig‐Haus Julius‐Maximilians‐University Würzburg Friedrich‐Bergius‐Ring 15 97076 Würzburg Germany

**Keywords:** bioprinting, fibers, filler materials, melt electrofibrillation, muscle alignment

## Abstract

Cellular alignment is essential for the function of anisotropic tissues such as skeletal muscle, tendon, cardiac, or neuronal tissues, where cell polarization governs mechanical integrity and signal transduction. However, engineering 3D tissue constructs with anisotropic extracellular microenvironments remains challenging, especially in larger constructs, which are commonly fabricated using extrusion‐based bioprinting of cell‐laden hydrogels, also known as bioinks. Here, a new class of bioprintable fibrous filler materials, fibrillar bundles, is presented that can be incorporated into bioinks and harness shear forces during extrusion bioprinting to achieve in situ alignment without the need for additional processing steps. These fibril bundles consist of multiple submicrometer fibrils fused into a larger bundle. They support robust cell adhesion and effectively promote polarization and alignment across multiple cell types. When incorporated into bioinks and printed with muscle cells, the fibrillar bundles enhance cellular alignment, and quantitative analysis confirms the directional growth of multinuclear myotubes and their morphological maturation. This approach offers a scalable and integrative solution for inducing anisotropy within 3D biofabricated tissues, holding promise for applications in muscle tissue engineering and beyond.

## Graphical abstract

Fibrillar bundles are introduced as a bioprintable additive that enables robust and scalable cellular alignment within 3D constructs through flow‐induced orientation during extrusion. These fibers support strong cell adhesion and polarization across various cell types and significantly enhance myotube alignment in Gelatine‐Methacryloyl (GelMA) hydrogels. This approach provides a versatile platform for engineering aligned, anisotropic tissues such as skeletal muscle.

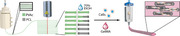

## 1 Introduction

Directionality of extracellular matrix (ECM) components is a prerequisite for cellular alignment and bio‐functionality of various tissues, such as cardiac,^[^ ^1^ ^]^ skeletal muscle,^[^ ^2^, ^3^ ^]^ smooth muscle,^[^ ^4^ ^]^ tendon,^[^ ^5^ ^]^ or nerve tissue.^[^ ^6^ ^]^ For instance, the axially aligned fibrillar structure of skeletal muscle tissue's ECM forms a bundle, which guides the cellular growth and muscle cell alignment in favor of myogenesis and unidirectional force generation.^[^ ^7^ ^]^ Similarly, in smooth and cardiac muscle, muscle fibers align in the direction of force generation, enhancing both electrical impulse transmission for synchronized contraction and mechanical stability for effective force transmission.^[^ ^1^, ^4^, ^8^ ^]^ With regards to tendon, collagen fiber anisotropy as well as resident tendon‐lineage cell organization in parallel rows in between the aligned fibers are key characteristics of a healthy tissue, and become disturbed in the course of tendinopathy.^[^ ^9^, ^10^ ^]^ In nerve regeneration, cell alignment is required to direct axonal outgrowth along anisotropic cues toward their targets, ensuring functional signal transduction and tissue integration.^[^ ^11^ ^]^ Taken together, these examples highlight that ECM anisotropy is a fundamental structural feature that supports both tissue architecture and function.

In tissue engineering, recapitulating such anisotropy in 3D models is essential to mimic these natural structures and guide directional cellular growth. Various approaches have been developed to provide alignment cues, including surface micro‐ and nanotopographies such as microgrooves,^[^ ^12^ ^]^ ridges,^[^ ^13^ ^]^ fibers,^[^ ^14^ ^]^ micropatterns,^[^ ^15^, ^16^, ^17^ ^]^ or pillars.^[^ ^18^, ^19^ ^]^ Fibrous substrates are especially attractive, and a range of fabrication technologies exists to generate fibers with different orientations, geometries, and porosities. Wet‐spun fiber bundles,^[^ ^20^ ^]^ electrospun meshes,^[^ ^21^, ^22^ ^]^ touch‐spun mats,^[^ ^23^ ^]^ and melt electrowritten grids^[^ ^24^, ^25^ ^]^ have all been employed to create 2.5D and 3D scaffolds. Melt Electrowriting (MEW)Good Manufacturing Practice is a technology that allows excellent deposition control of fibers with complex patterns and controlled topography; however, it provides overall lower throughput, producing small fiber diameters compared to electrospinning and touch spinning techniques.^[^ ^26^ ^]^ A new derivative of MEW combines the deposition precision of MEW with the ability to generate continuous, small‐diameter fibrillar fibers through flow‐directed polymer phase separation, a process known as melt electrofibrillation (MEF).^[^ ^27^, ^28^ ^]^ Developing functional tissue models for applications such as personalized disease models,^[^ ^29^ ^]^ drug testing,^[^ ^30^ ^]^ or tissue replacements^[^ ^31^ ^]^ lies in fabricating 3D structures that incorporate hierarchical architectures with distinct cell‐ and tissue‐specific cues. Extrusion‐based bioprinting has gained significant interest for producing 3D constructs by depositing cells embedded in hydrogels, so‐called bioinks.^[^ ^32^ ^]^ This approach also enables the precise spatial placement and combination of different cell types within a single tissue construct, similar to native tissues, to gain functionality.^[^ ^33^, ^34^ ^]^ Moreover, bioinks can be further functionalized by incorporating additional fillers, providing features such as controlled drug delivery,^[^ ^35^ ^]^ mechanical reinforcement,^[^ ^36^ ^]^ enhanced biological interactions, anisotropy, and cellular directionality in the 3D printed structures.^[^ ^37^, ^38^, ^39^ ^]^

Several strategies have been explored to introduce fibrous architectures into hydrogels during or after printing. For example, fibrous patterns can be formed in situ by selectively crosslinking certain regions, as in the filamented light (FLight) approach,^[^ ^40^, ^41^ ^]^ or by aligning collagen fibrils during extrusion.^[^ ^42^ ^]^ Alternatively, pre‐formed fibers can be incorporated into the hydrogel as filler materials.^[^ ^39^, ^43^ ^]^ Such fibers not only enhance cell alignment but also improve print fidelity and mechanical performance.^[^ ^44^ ^]^ Post‐deposition alignment methods, including magnetic^[^ ^45^, ^46^ ^]^ or acoustic fields,^[^ ^47^ ^]^ as well as flow‐driven orientation in the nozzle during printing caused by extensional flow,^[^ ^43^, ^48^, ^49^ ^]^ further expand the range of alignment strategies. To ensure the printability of such composite bioinks, the fibrous filler length and density must be limited to prevent entanglement and nozzle clogging during extrusion.^[^ ^50^ ^]^ Conventional fiber fragmentation techniques often suffer from low yield or inconsistent fiber lengths.^[^ ^3^, ^51^, ^52^, ^53^ ^]^ We recently addressed this by developing a cryo‐cutting workflow for producing short, precisely controlled fibers from a range of materials, stabilized on water‐soluble adhesive substrates during collection and processing.^[^ ^48^ ^]^ This enables the targeted design of short fibers that can be aligned along the extruded strand during 3D printing.

Despite these advances, a critical limitation persists: short fibers produced from synthetic polymers such as poly(lactic‐co‐glycolic acid) (PLGA)^[^ ^3^ ^]^ and poly(ε‐caprolactone) (PCL)^[^ ^48^, ^54^ ^]^ suffer from low cell adhesion; therefore, to be used as cell carriers within the printed structure, additional post‐treatment, e.g., plasma,^[^ ^55^ ^]^ or immobilization of the proteins is required.^[^ ^56^ ^]^ If these short fibers are cell‐adhesive and the hydrogel matrix of bioink permits sufficient cellular mobility, they can be used to induce intra‐strand cellular alignment, a feature that is essential for creating biofunctional tissue models with high anisotropy and cellular directionality.^[^ ^43^, ^49^ ^]^ As a result, the development of fibrous fillers that facilitate good cell‐adhesion and can align with extrusion‐based bioprinting remains an unmet need.

In this study, we present a new strategy to address these challenges by fabricating fibrillar bundles using the MEF, employing PCL as the hydrophobic, water‐insoluble structural polymer and a hydrophilic, water‐soluble porogen, to induce phase separation and generate fibrillar micro‐structures.^[^ ^27^, ^57^ ^]^ Building on this approach, we developed a printable composite bioink incorporating laser‐cut MEF fibrillar bundles, designed to align under extensional flow during extrusion and to guide cellular organization within printed strands. Due to their morphological features mimicking collagen‐fibrillar topography, these MEF‐derived bundles are intrinsically cell‐attractive without requiring post‐treatment, enabling them to act directly as adhesive carriers within bioinks. To assess their influence, we investigated how these aligned fibrillar bundles affect the orientation of various cell types embedded in the hydrogel. As a model system, we encapsulated C2C12 skeletal muscle cells and analyzed their interactions with the fibrous architecture during the growth phase and myogenesis.

## 2 Results and Discussion

### 2.1 Fabrication and Cutting of Fibrillar Bundles

**Figure 1:**
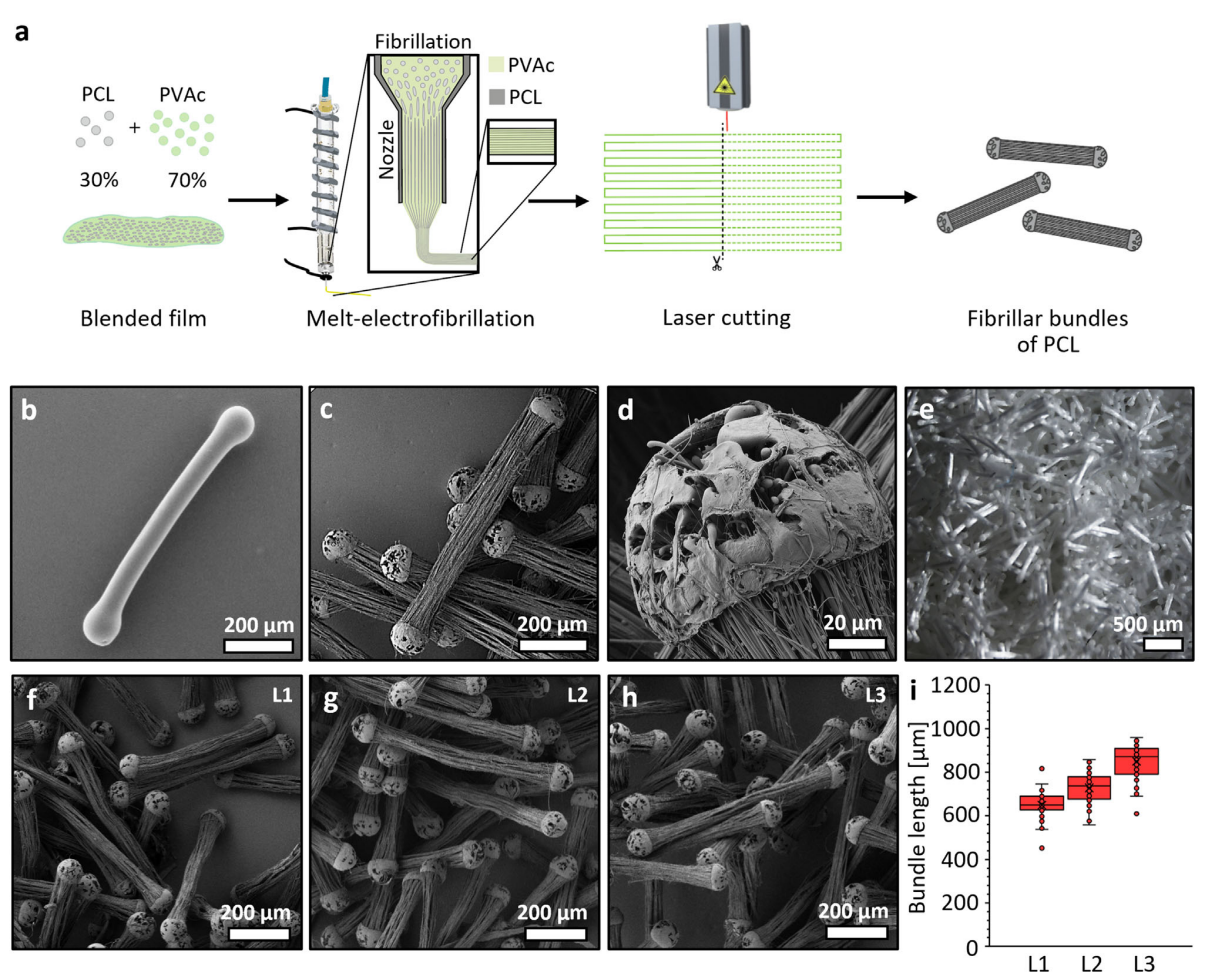
Production of fibrillar bundles. a) A schematic drawing presents the fabrication steps and fragmentation of fibrillar bundles. SEM images of a laser‐cut fibrillar bundle, b) before removal of the PVAc matrix, and c) after its removal by washing in 70% ethanol. d) A close‐up of the fused molten end of the cut bundles. e) Freeze‐dried pellet of fibrillar bundles. f–h) SEM images and the fibrillar bundles produced with lengths around f) L1 600 µm, g) L2 700 µm, h) L3 800 µm using different arrays (n=50), i) box plot of the measured lengths of the fibrillar bundles.

In an earlier study, we showed that using a blend of 30% (w/w) PCL and 70% (w/w) poly(vinyl acetate) (PVAc), continuous fibrillar bundles could be fabricated under defined conditions in the MEF processing.^[^ ^27^ ^]^ Before the MEF process, both a water‐insoluble PCL and a water/ethanol soluble PVAc were co‐dissolved, cast as a film, and, after solvent evaporation (dichloromethane), melted and processed via MEW. During this step, the hydrophilic polymer forms a continuous matrix, while the hydrophobic polymer forms a dispersed phase that is stretched into fibrils by extensional flow. Subsequent selective removal of the PVAc matrix by 70% ethanol yields a porous, aligned PCL fibrillar architecture (**Figure** **1**a).^[^ ^27^ ^]^ The specific blend of 30/70 PCL/PVAc was identified as the most suitable candidate for this novel approach. It yields an adequate density of PCL fibrils within the scaffold to form fibril bundles that confer stiffness and shape stability, while simultaneously maintaining a loosely organized fibrillar architecture with sufficient interstitial space to support cell attachment and promote cellular alignment, thereby mimicking structural elements of native extracellular matrices (ECM).^[^ ^27^, ^58^ ^]^

The fabrication of a composite bioink containing MEF fibril bundles for extrusion‐based bioprinting requires cutting the continuous fibril bundles to an adequate length to avoid entanglement and clogging the printing nozzle.^[^ ^59^ ^]^ Unlike conventional fibers, the fibrillated bundles cannot simply be cut or fragmented, as they would dissociate into single fibrils and thus lose their intended structural integrity as a bundle. Therefore, we applied a simple yet effective strategy for segmenting continuous 30/70 PCL/PVAc blend fibers using a laser cutter (Figure 1a). This approach enabled the simultaneous cutting of melt‐electrowritten fibers into uniformly sized segments, while melting the fiber ends, resulting in the fusion of fibrils into rounded cap fragments that hold the bundle together (Figure 1b–d). To attain consistently well‐fused fibrils at the ends, the fibril bundles had to be cut and suspended in the air. Therefore, arrays with regularly spaced gaps were used (Figure S1a, Supporting Information), on which the continuous fibers were printed perpendicularly to the gaps and then laser cut (Figure 1b). After cutting, the fragments could be removed from the grids by submersion in 70% ethanol, concurrently removing the PVAc fraction of the fibers and unveiling the fibrillar bundle structure of the cut fibrillar bundles (Figure 1c). The delicate fibrillar structure of the bundle fibers did not exhibit any damage during removal and was well defined and identical to uncut bundle fibers. Figure 1d presents the cut fibrillar bundle ends, rounded, dumbbell‐shaped, and porous, that resulted from the dissolution of PVAc. The fibrillar bundles could subsequently be freeze‐dried for weighing and storage without being damaged (Figure 1e). The fused caps of bundles containing the microfibril end impede the agglomeration of the bundles. However, in longer bundles, the microfibrils demonstrate a propensity to aggregate due to looser fibrils entangling (Figure 1g,h). While a longer fibrillar bundle with a larger surface area would be advantageous in attracting and carrying a higher number of cells, its length is a limiting factor for extrusion printing of the composite bioink.^[^ ^60^ ^]^ Therefore, we fabricated fibrillar bundles with different lengths (between 600 and 800 µm) to explore their stability and minimal aggregation in solution, as well as suitability for 3D printing with minimal nozzle clogging (Figure 1f,i). The fibers in each group showed a relatively narrow size distribution (Figure 1i; Figure S1b, Supporting Information).

### 2.2 Influence of Bioink on Fibril Bundles’ Alignment

To induce directional anisotropy in the 3D printed tissue models, the ability to align bundle fragments during extrusion printing was investigated. Prendergast et al.^[^ ^60^ ^]^ have shown that the flow profile in the nozzle can direct and align short fibrous fillers in the printing direction. Nonetheless, in our previous studies using predictive modeling, we showed that the viscosity of the hydrogel matrix, the density of the fillers in the ink, the ratio of the needle diameter to the filler diameter, and the subsequent spreading of the printed strut play a crucial role in fiber alignment.^[^ ^50^, ^61^, ^62^ ^]^ Therefore, a Pluronic‐based ink with different concentrations was used as an initial model bioink, given its well‐established printability and shape fidelity.^[^ ^61^ ^]^ The concentration‐dependent viscosity of the ink plays a critical role in ensuring that the fibril bundle fragments remain homogeneously dispersed within the hydrogel and are co‐extruded during printing.

**Figure 2:**
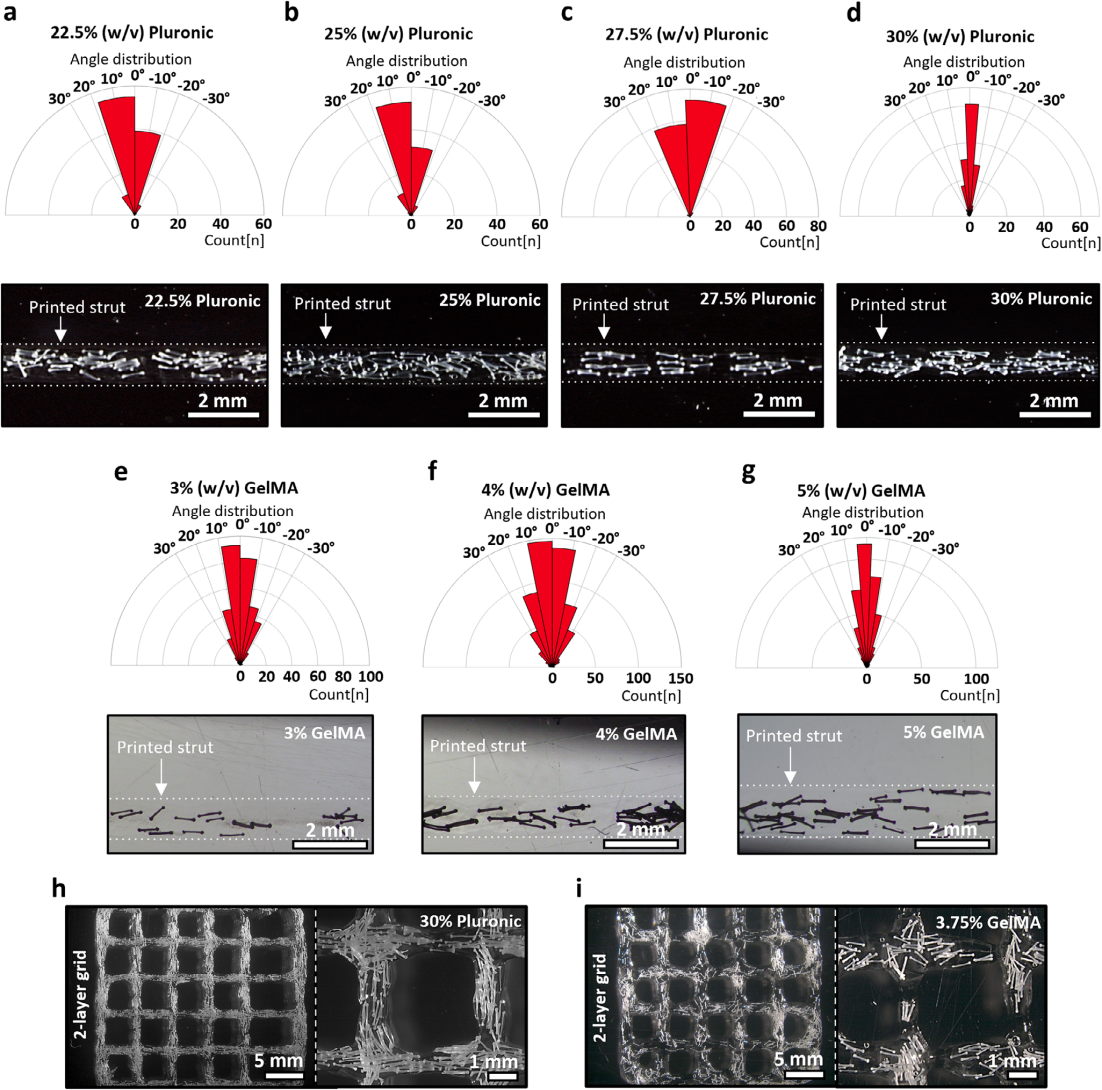
Alignment of fibrillar bundles after 3D printing. Quantification of the alignment and corresponding exemplary images of fibrillar bundles after extrusion‐printing at a 3% (w/v) bundle concentration in Pluronic at a polymer concentration of a) 22.5%, b) 25%, c) 27.5 and d) 30% (w/v) (n=100), as well as for the same concentration of fibrillar bundles printed in e) 3%, f) 4% and g) 5% (w/v) GelMA concentrations (n=350), as well as 2 layer grid structures printed with h) 30% Pluronic and i) 3.75% GelMA each containing 3% fibers bundles.

Printing was conducted at a constant speed of 10 mm s^−1^ using a G18 conical nozzle, with a printing pressure ranging between 0.2 and 0.38 bar depending on concentration. At higher Pluronic concentrations of 22.5–30% (w/v), the viscosity was sufficient to maintain a uniform distribution of 3% bundles and to generate the necessary shear and extensional forces to align and transport them through the nozzle (**Figure** **2**a–d). The measured alignment within a ±5° deviation from the printing direction showed an increasing trend as the Pluronic concentrations were raised to 27.5%, with alignment values of 37.4% ± 11.3% for 22.5% Pluronic, 40.5% ± 7.8% for 25%, and 59.8% ± 7.2% for 27.5% and 54.2% ± 22.3% (Figure S2c, Supporting Information). This trend could also be deduced from the resulting alignment within a ±10° deviation from the printing direction, showing an increasing trend of alignment with higher Pluronic concentrations to 60.1% ± 6.8% for 22.5% Pluronic, 62.6% ± 7.3% for 25% Pluronic, 81.8% ± 9.6% for 27.5% Pluronic, and 77.2% ± 8.6% for 30% Pluronic (Figure 2a–d). Even though a trend was visible, the concentration of Pluronic did not significantly influence the bundle alignments within the ±5° or ±10° deviation of printing direction (Figure S2d, Supporting Information). The slight trend toward improvement in alignment can be attributed to the increased viscosity and, consequently, stronger flow‐induced alignment forces. In contrast, at concentrations of 20% (w/v) and below, the viscosity was not sufficient to prevent premature agglomeration of the fibril bundles within the cartridge. This led to an inhomogeneous distribution, hindering co‐extrusion and resulting in nozzle clogging or incomplete filler deposition (Figure S2a, Supporting Information). Overall, a sufficiently high viscosity is crucial not only to prevent agglomeration but also to maintain a stable dispersion and to promote flow‐induced fibril bundle alignment during extrusion.

Since Pluronic is unsuitable for cell culture, lacking cell‐adhesive motifs and an appropriate pore size to support cell spreading or proliferation, Good Manufacturing Practice(GMP)T‐grade gelatin methacryloyl (GelMA) was used instead for subsequent cell alignment experiments. To achieve proper fiber alignment in the needle, comparable to Pluronic, GelMA must show thixotropic behavior, comparable recovery behavior, and sufficient viscosity. Increasing shear stresses correlate to higher alignment, but if too high, they can reduce cell viability and function. Differences in GelMA concentration affect viscosity and consequently shape fidelity, and bioink shear recovery.^[^ ^44^ ^]^ In this regard, GelMA, with a concentration range of 3–5% (w/v), exhibiting shear‐thinning behavior, was used to investigate the alignment of 3% (w/v) fibrillar bundles during extrusion printing of composite ink, at a constant speed of 10 mm s^−1^, and 0.38 bar, using a G18 conical nozzle as with the Pluronic (Figure 2e–g). The calculated wall shear stress for the GelMA ink with 3% bundle fibers was 2.5 kPa, well below the 5 kPa threshold known to negatively affect cell viability.^[^ ^63^ ^]^

**Figure 3:**
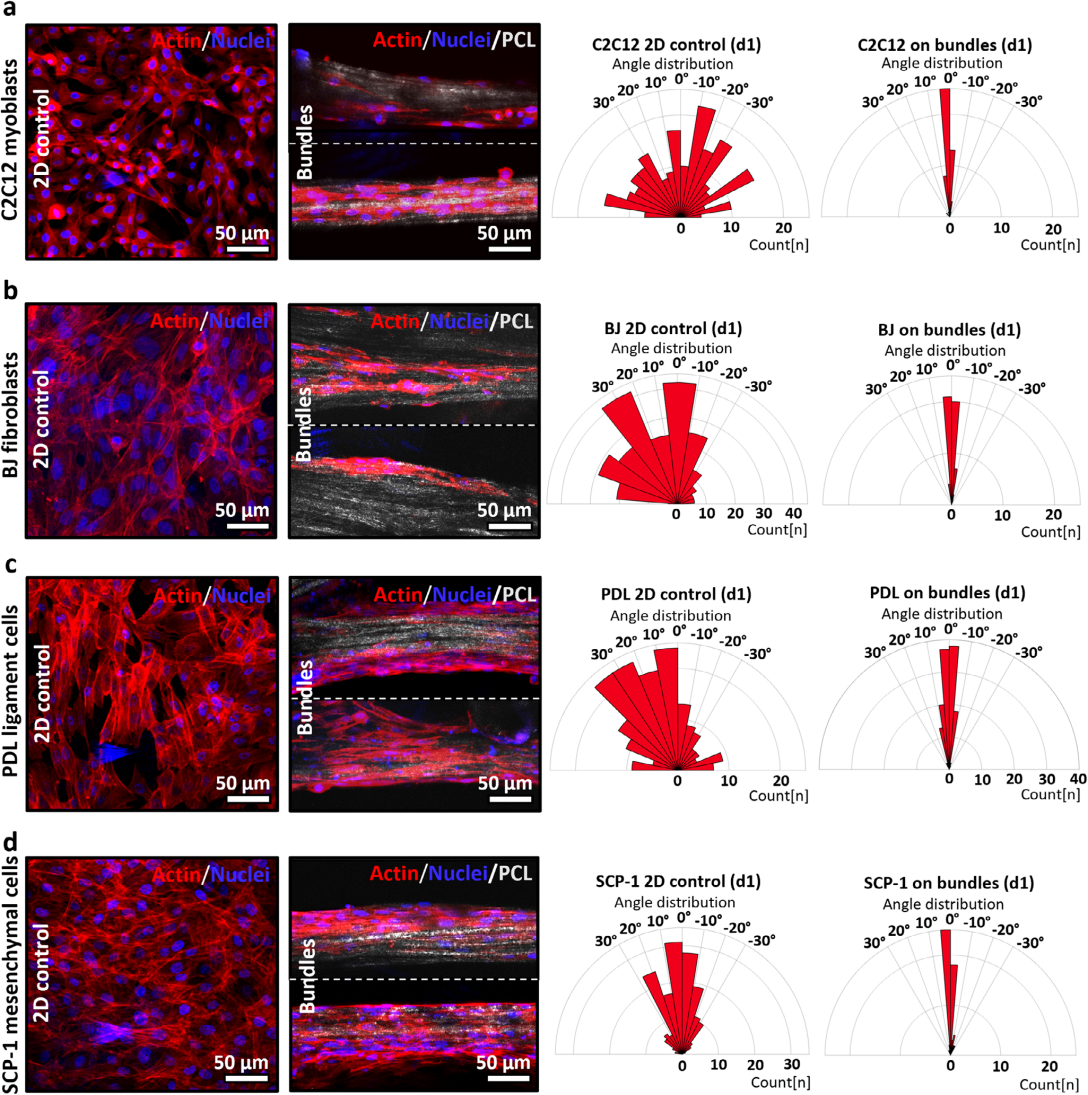
Cellular alignment of different cell lines imaged by confocal microscopy after staining the cytoskeleton, actin filaments, and nuclei using Phalloidin‐iFluor 555 and Hoechst in red and blue, respectively. Cells on flat polystyrene tissue culture plate substrates are used as a control and compared with fibrillar bundles after one day of culture. The alignment of the cells based on a ±5° deviation of their cytoskeleton from the axis of the fragments was analyzed and plotted for a) C2C12 murine myoblasts, b) BJ‐TERT human fibroblasts, c) PDL‐hTERT immortalized human periodontal ligament progenitor cell line, and d) SCP‐1‐TERT human mesenchymal bone marrow‐derived stem cell line (n=50).

The alignment of fibers within a ±5° deviation from the printing direction reached 32.2% ± 7.2% for 3% GelMA, 37.8% ± 8.1% for 4% GelMA, and 34.1% ± 9.1% for 5% GelMA (Figure S2e, Supporting Information). Respectively, 54.8% ± 8.3% for printed bundles within a 3% GelMA ink, 61.2% ± 10.6% in a 4% GelMA ink, and 58.8% ± 6.1% in a 5% GelMA ink are oriented within ±10° of the printing direction (**Figure** **3**a). However, none of the different tested GelMA concentrations were significantly different from each other in terms of alignment (Figure S2f, Supporting Information).

The shear thinning properties of Pluronic and GelMA have already been reported in the literature;^[^ ^61^, ^64^, ^65^ ^]^ however, the viscosity and recovery behavior of the inks strongly depend on the concentration, molecular weight, and extrusion temperature. Therefore, a rheological recovery test was done to compare the gel's properties during and after extrusion printing. The viscosity of Pluronic at 0.1 1/s shear rate is 8.9 ± 0.25 kPa for 30%(w/v), 3.6 ± 0.07 kPa for 27.5%, 1.7 ± 1.45 kPa for 25% and 1.1 ± 0.05 kPa for 22.5%. At a higher shear rate of 1 1/s, the viscosity of Pluronic decreased to 472.0 ± 19.60 Pa for 30%, 429.9 ± 0.42 Pa for 27.5%, 345.5 ± 2.31 Pa for 25% and 141.0 ± 2.83 Pa for 22.5% (Figure S3a, Supporting Information). The viscosity of GelMA for all tested concentrations at low shear rates was much lower compared to Pluronic. GelMA at its processing temperature of 23 °C, and at a shear rate of 0.1 1/s, resulted in a viscosity of 0.7 ± 0.18 kPa for 5%, 0.3 ± 0.03 kPa for 4%, 0.13 ± 0.01 kPa for 3.75% and 60.1 ± 6.91 Pa for 3%. With increasing shear rate to 1 1/s, GelMA's viscosity dropped to 67.1 ± 3.43 Pa for 5%, 26.3 ± 1.08 Pa for 4%, 15.0 ± 0.81 Pa for 3.75% and 6.98 ± 0.4 Pa for 3% (Figure S3b, Supporting Information). The recovery test showed that varying the concentration of Pluronic and GelMA did not influence the speed of recovery for each of the inks. Pluronic generally recovers from high shear rates faster than GelMA; however, both inks showed good recovery behavior after high stress loading (Figure S3, Supporting Information), as presented also by Jongprasitkul et al.^[^ ^66^ ^]^

When printed in more complex two‐layer grid patterns using Pluronic or GelMA (Figure 2h,i), both materials were successfully deposited, and the fibers remained visibly aligned. The Pluronic construct exhibited a more uniform appearance, which was also the case during the previously discussed alignment experiments. This is due to the comparatively better printing performance of Pluronic, which, among others, also has a higher viscosity compared to the GelMA across all concentrations (Figure S3a,b, Supporting Information). These viscosity differences, therefore, result in better deposition, easier extrusion of the fibers, and less strut spreading after deposition for Pluronic composite ink, which is in agreement with the previous studies.^[^ ^61^, ^67^ ^]^

When attempting to use bundle concentrations exceeding 3% (w/v), it resulted in decreased alignment due to an increased bundle‐bundle interaction and formation of loose clumps (Figure S2b, Supporting Information), independent of the GelMA concentration. Although higher fiber concentrations could potentially improve cell alignment, the fiber concentration was restricted to 3% (w/v) in GelMA. Given their large size, low concentration, and loose distribution, the fibers were unlikely to reinforce or stabilize the hydrogel. This was acceptable, as the objective of the study was to induce cellular alignment rather than to enhance the mechanical properties of the bioink. Since previous studies had shown uninhibited cell spreading up to 3.75% (w/v) GelMA concentrations^[^ ^68^, ^69^ ^]^ and we achieved adequate fiber alignment within the 3‐5% range with slightly improved printability at higher GelMA concentrations, we selected a 3.75% concentration for subsequent experiments and cell culture studies.

### 2.3 Cellular Interactions with Fibril Bundle Fragments in 2D

After identification of sufficient parameters to induce fibrillar bundle alignment upon 3D printing, we evaluated the interaction of the fibrous component of the composite bioink with cells to determine its ability to guide cellular growth and alignment along the fiber axis. This is pertinent for tissues in which cell growth and migration directionality are of high importance.^[^ ^2^ ^]^ Accordingly, to validate the concept, several representative cell lines were systematically assessed for their ability to adhere to the fibrillar bundles in 2D and align along them in comparison to the tissue culture plate substrate used as a control to demonstrate how the cells would otherwise behave without any directional cues.

The cell lines used were human immortalized fibroblasts (BJ‐TERT), murine skeletal muscle cells (C2C12), human immortalized periodontal ligament progenitor cell line (PDL‐hTERT), and a human mesenchymal bone marrow‐derived stem cell line (SCP‐1‐TERT). Human telomerase reverse transcriptase (hTERT)–immortalized primary cells are ATCC genetically modified human primary cells that exhibit the growth characteristics of a continuous cell line with extended proliferative capacity yet maintain the physiology of a primary cell.

As could be deduced by seeding the cells onto the bundles and monitoring their interactions with the bundles for a period of 1–3 days (**Figure** **3**; Figure S4, Supporting Information), all the cell lines attached to the fibril bundles. Furthermore, the cells in the culture proceeded to adhere, elongate, and migrate along the bundle orientation. After 1 day of culture, (Figure 3) the highest degree of cellular alignment was measured at ≈94.6% ± 6.8% within a ±10° deviation from the fiber axis for BJ fibroblast cells, followed by 93.4% ± 8.6% alignment for C2C12 muscle cells, 88.4% ± 8.6% for PDL‐hTERT cells, and 83.5% ± 1.6% for SCP‐1‐TERT stem cell line, clearly indicating that all cells aligned well along the fibrils, whilst they exhibited a random orientation in the control without directional cues. The differences in alignment between different types remained insignificant (Figure S3a, Supporting Information). On the third day, the alignment was maintained high, with no significant difference in alignment from day 1 (Figure S3b–e, Supporting Information). Noteworthy, this degree of alignment and cell affinity for the bundle fibers was achieved without any additional ECM‐based coatings or functionalization, which are commonly employed to promote cell adhesion and guidance.^[^ ^39^, ^48^, ^70^ ^]^ This suggests a strong impact of the native‐like submicrometer‐scale fibrillar architecture of the bundles, which provides cells with sufficient biophysical cues to facilitate fiber grasping and stable attachment.^[^ ^71^ ^]^ This behavior can be attributed to the bundles’ ECM‐mimicking topography, characterized by a high surface area, appropriate fibril diameter and spacing, and open porosity.^[^ ^27^, ^72^, ^73^, ^74^ ^]^ The average diameter of the fibril bundles is 60 ± 10 µm (Figure S1b, Supporting Information), whereas fibrils within the bundles exhibit an average diameter of 400 ± 430 nm (Figure S1, Supporting Information). This broad distribution likely arises from the intrinsic variation in PCL droplet size within the polymer blend melt and the partial fusion of droplets during elongation.^[^ ^75^ ^]^ Nonetheless, most fibrils remain in the lower submicrometer range, closely resembling collagen I fibrils with diameters of ≈100–300 nm.^[^ ^76^ ^]^ According to Gao et al.,^[^ ^71^ ^]^ fiber diameters in the range of 2.48–18.3 µm offer a cell‐favorable microenvironment, but may lack sufficient strength to keep the shape fidelity under high cell loading. In our case, forming a bundle structure of thin fibril fibers offers this missing mechanical support to the cells while maintaining the favorable micro‐architecture.

### 2.4 Myoblast Alignment and Maturation on Bundles in 2D

**Figure 4:**
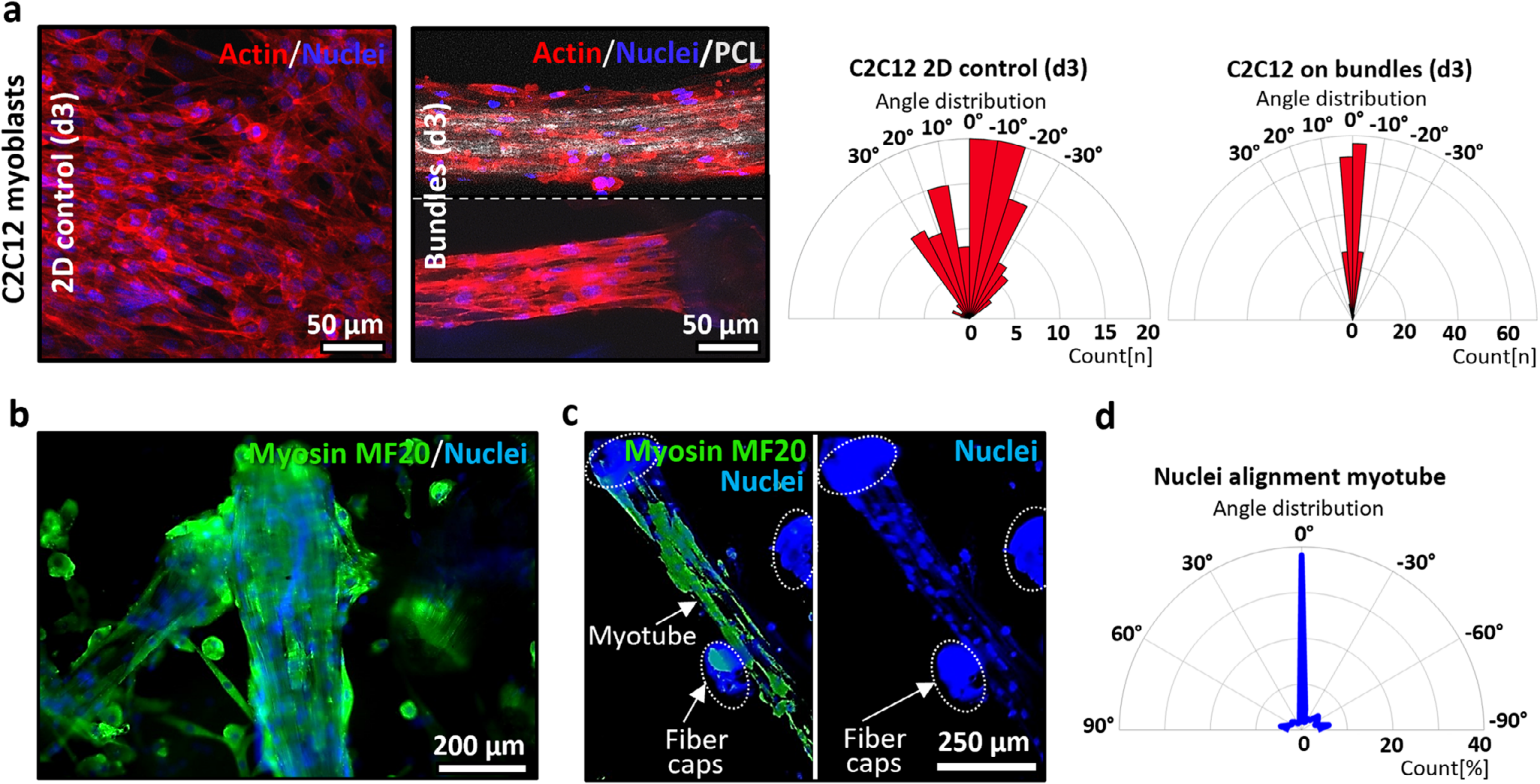
Muscle cell alignment and myogenesis on fibrillar bundles. a) Fluorescence images of C2C12 murine myoblast cells cultured on cell adhesive well plates as a control and on fibrillar bundle fibers for three days, showing their cytoskeleton and nuclei (Phalloidin‐iFluor 555 and Hochest were used to stain the actin filaments and nuclei in red and blue, respectively). The cellular alignment was performed based on a ±10° deviation of their cytoskeleton from the axis of the fragments (n=180). b) Confocal microscopy image of a fibrillar bundle fully covered by differentiated myotubes after three days of culture in proliferation medium and four days of differentiation. The myotubes are stained for fast and slow myosin 4 monoclonal primary antibody (MF20), revealed by donkey anti‐mouse IgG secondary antibody, Alexa Fluor Plus 647. DAPI was used to stain nuclei and imaged at 20x magnification. c) Confocal microscopy image of a fibrillar bundle with MF20‐stained 10‐nuclei‐long myotube in 20x magnification, and d) Quantification of the myotubes' nuclei alignment and the deviation from the axis of the fiber bundle (n= 180).

Skeletal muscle is one of the tissues with high anisotropy in which adequate initial cell alignment is vital for further maturation of the cells and later the attainment of the natural function of muscle tissue in directed contraction and movement of the skeletal body.^[^ ^2^ ^]^ As shown in Figure 3a, the C2C12 myoblast cells already displayed a high cell alignment after one day of culture on fibrillar bundles in 2D. Continuing culturing the cells for three days, we observed that their alignment was maintained despite the increased cell density resulting from proliferation (**Figure** **4**a). The contact guidance impact of the fibrillar bundles was also proven during the differentiation of the myoblasts and formation of multinuclear myotubes. During myogenesis, myoblasts undergo genetic reprogramming and metabolic shifts. Activated myoblasts rely on anaerobic glycolysis to support rapid biosynthesis and proliferation. As differentiation progresses, mitochondrial biogenesis is upregulated to meet the energy demands of contraction.^[^ ^77^ ^]^ Upon fusion into multinucleated myotubes, cytoplasmic volume and mitochondrial density increase in response to growing myotube width.^[^ ^78^ ^]^ The C2C12 cells on fibril bundle fibers could differentiate into long myotubes after 7 days of culture (3 days in proliferation followed by 4 days of differentiation) (Figure 4b), which completely covered the whole bundle surface with their fibrillar topography. The fibrillar bundles helped facilitate the formation of aligned myotubes with up to nine nuclei (Figure 4c). Furthermore, the width of the elongated myotubes on the PCL fibril bundle fibers was measured with an average of 28 ± 8.7 µm, which is similar to our previous study culturing and differentiating the C2C12 cells on wet‐spun collagen fibers with microfibrillar morphologies.^[^ ^79^ ^]^ The alignment of myotubes on fibril bundles was calculated based on the elongation of the nuclei of the cells along the axis of the fibers. Orientation analysis using OrientationJ revealed that 71% ± 0.2% of the nuclei were aligned within a ±5° deviation of the bundle orientation (Figure 4d; Figure S3c, Supporting Information). The remaining ≈30% represent myotubes that either span across multiple bundles or interact with the underlying surface. The calculated myotube alignment on fibrillar bundles, with a ±10° deviation, compares favorably to previous studies. For instance, Narayanan et al.^[^ ^80^ ^]^ reported that myotube nuclei alignment cultured on electrospun PLGA fibers was ≈38% after, while Choi et al.^[^ ^81^ ^]^ reached 80% with ±10° deviation on electrospun collagen‐PCL fibers. Higher myotube alignment >90% was reported by Wang et al.^[^ ^82^ ^]^ using wet electrospinning PCL–silkfibroin–polyaniline fiber yarns, as well as Koeck et al.^[^ ^20^ ^]^ using wet‐spun collagen fibers type I/II with microfibrillar structures. It must be mentioned that the given examples used continuous fibers for differentiating the C2C12 cells, and the alignment of myotubes on short fibers or fragments can be influenced by the limited available space for cells to grow.

### 2.5 Bioprinting of Myoblast‐Laden Composite Bioinks

**Figure 5:**
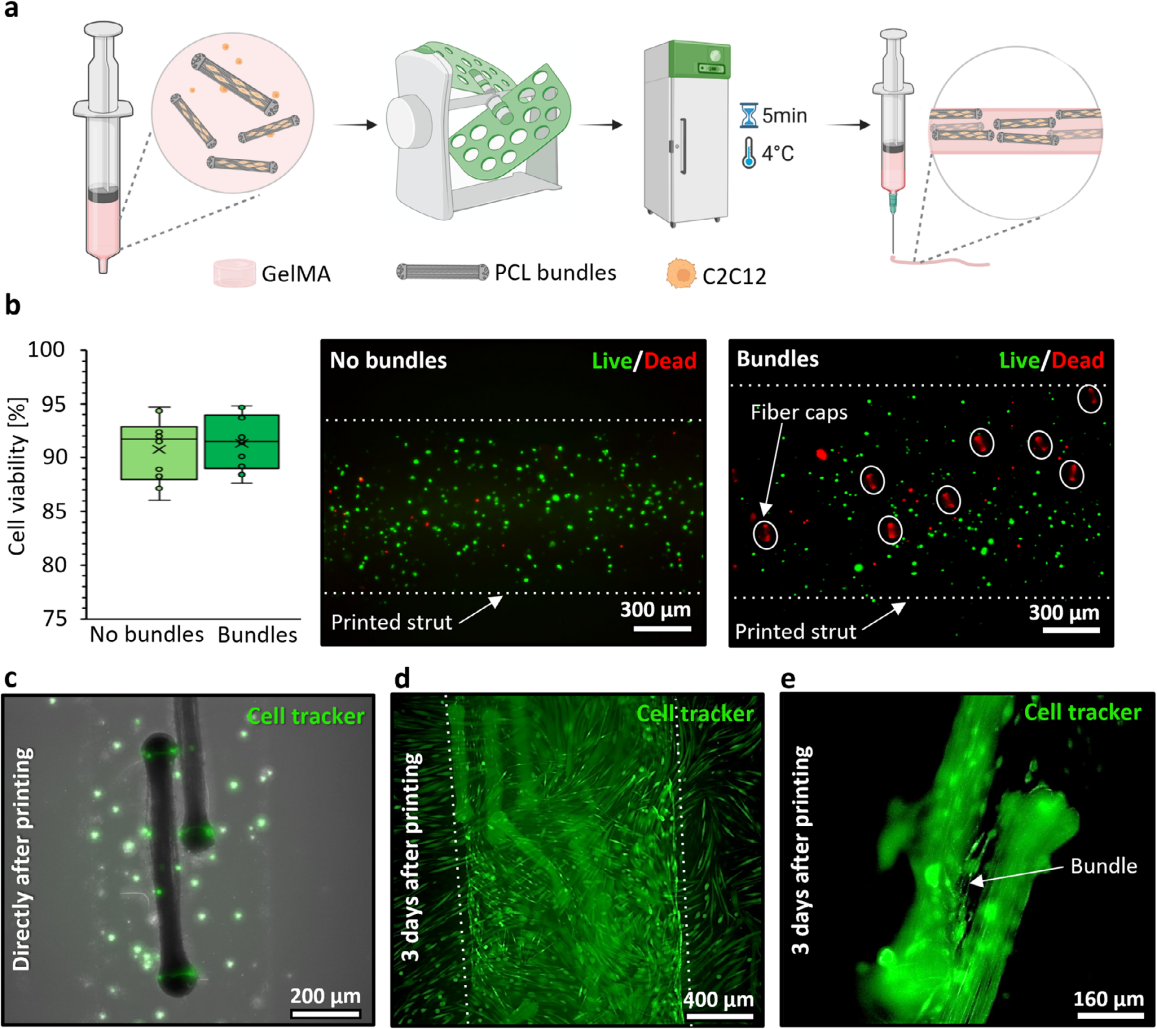
Bioprinting composite bioinks containing encapsulated C2C12 myoblast cells in GelMA with 3.75% concentration. a) Schematic representation of GelMA composite bioink preparation. b) Quantification of the cell viability after 3D bioprinting of the composite bioink using live/dead assay and fluorescent microscopy imaging (*calcein* AM (green) and *ethidium* homodimer‐1 (red)) with and without the fibrillar bundles. Fiber caps are spotted with red color due to the autofluorescence. Monitoring the cell behavior using green cell tracker‐stained myoblasts and fluorescence microscopy images (n=3), c) directly, and d) three days after 3D bioprinting. e) a larger snapshot of the fully cell‐covered bundle fiber cultured for three days after 3D bioprinting.

Bioprinting of the composite bioink composed of myoblast cells and 3% of fibrillar bundles as fillers mixed with a 3.75% GelMA hydrogel was investigated to evaluate the interaction of muscle cells and bundles as a 3D tissue culture model (**Figure** **5**a). First, the viability of the cells during extrusion printing in the presence of fibrillar bundles was assessed to ensure adequate printing parameters for maintaining the cells’ viability. Therefore, the prepared bioinks were printed as straight lines containing 0.5 million cells mL^−1^ with and without bundles and crosslinked immediately after printing. A live/dead assay was conducted directly after 3D printing (Figure 5b). Comparing bioinks with and without fillers, we observed that cell viability was not significantly different (91% ± 2.4% viable cells in the bioink containing fibril bundle fibers compared to 91% ± 2.8% viable cells in the bioink without bundles). Noticeably, the ends/caps of the bundles showed a consistently intense red autofluorescence signal in this assay, as it was also observed in other fluorescence‐microscopic investigations thereof, and were hence ignored. Second, to further investigate the cell interactions with the fibrillar bundles during 3D printing, we labeled 0.5 million cells mL^−1^ with a cell‐tracker prior to printing and mixing with the bundles. Figures 5c–e proved that pre‐culture of C2C12 with the bundle fibers before 3D printing is not necessary. Monitoring the cells encapsulated in the composite bioink, for a period of three days of culture, revealed that the cells are able to migrate toward and onto bundles. They use the microfibrillar topography to crawl up, adhere, spread, and align, as was shown already in Figure 3. During the longer culture time in the growth medium, cells can proliferate, further migrate along their curvature, and cover the fiber bundles. The proliferation and coverage of the fibrillar bundles after three days of culture are shown in Figure 5c,d, where the initially black‐appearing fibril bundle fibers turn green due to the presence of the fluorescently labeled cells.

### 2.6 Myogenesis Analysis on 3D Bioprinted Composite Bioink Containing Fibrillar Bundles

**Figure 6:**
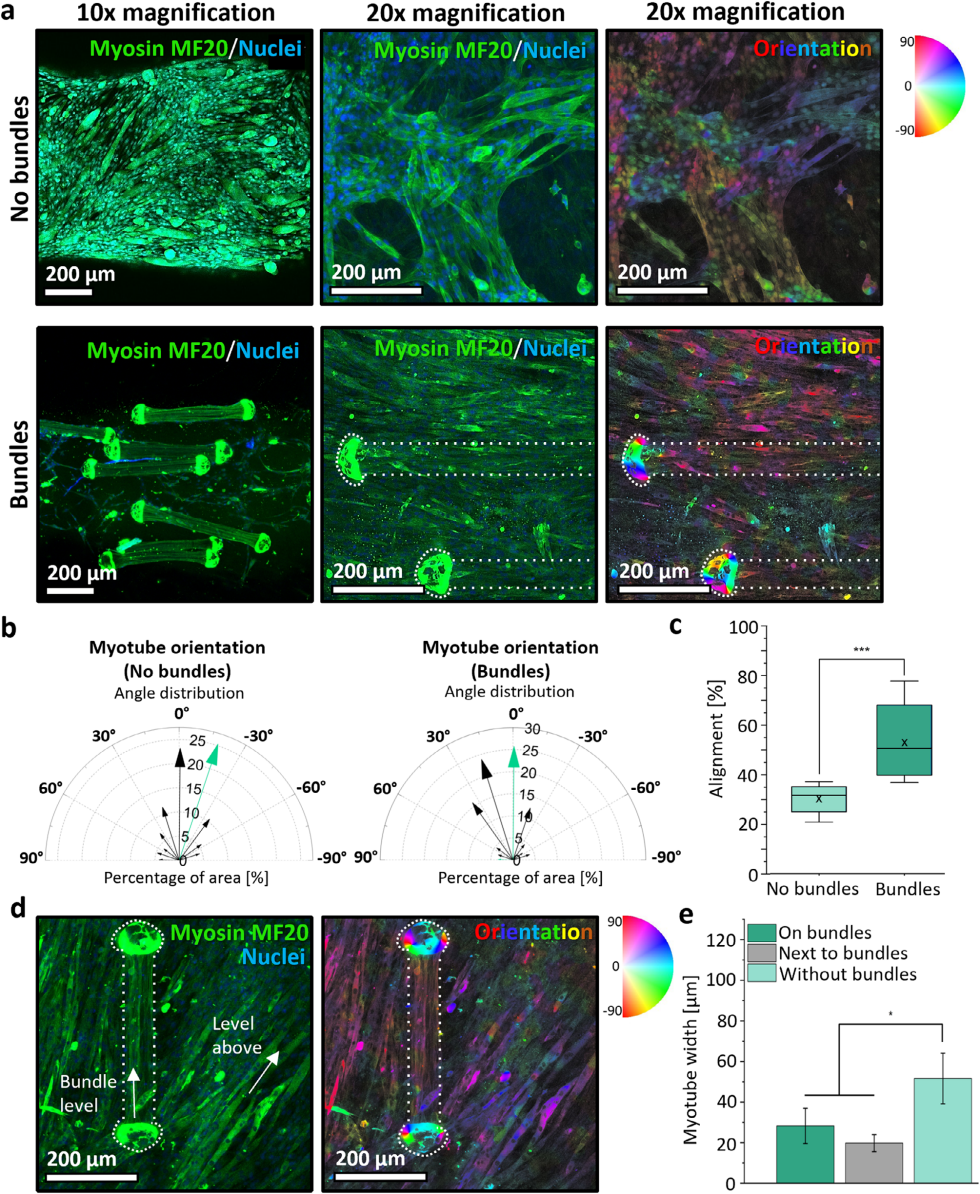
Myotube orientation analysis after 3D bioprinting of the bioink (3.75% GelMA and 3% fiber bundles), cultured for 3 days in growth medium and 4 days of differentiation. a) Confocal microscopy images of differentiated myotubes stained for slow and fast myosin heavy chain without (top) and with fibrillar bundles (bottom) in 10x (left) and 20x (middle) and as a color‐coded orientation map in 20x (right). The myotubes are stained for fast and slow myosin 4 monoclonal primary antibody (MF20), revealed by donkey anti‐mouse IgG secondary antibody, Alexa Fluor Plus 647. DAPI was used to stain nuclei. b) Compass plot of aligned myotube area (%) within ±20° deviation from the central 90° orientation, comparing printed constructs without (left) and with fibrillar bundles (right). The green arrows indicate the predominant direction of myotube growth. c) Quantification of myotube orientation distribution in GelMA with and without bundles with ±20° deviation (*p*< 0.001 = ^***^). d) Confocal microscopy images of differentiated myotubes stained for slow and fast myosin heavy chain (MF20) in different z‐areas, with bundle fibers in 20x magnification (left) and as a color‐coded orientation map (right). The myotubes are stained for myosin 4 monoclonal primary antibody (MF20), revealed by donkey anti‐mouse IgG secondary antibody, Alexa Fluor Plus 647. DAPI was used to stain nuclei. e) Quantification of myotube width (µm) growing on bundles, next to bundles, and without bundles.

To assess the impact of the fibrillar bundles on myogenesis of encapsulated C2C12 myoblasts and myotube orientation within the GelMA 3D printed construct, the bioink was printed and cultured for three days in proliferation medium, followed by four days in supplemented differentiation medium. The myotubes were stained for the myosin heavy chain (MF20), revealing a high density of differentiated cells in the GelMA‐based bioink without bundle fibers (**Figure** **6**a top) and in the presence of 3% bundle fibers in the bioink, growing around and alongside the bundles (Figure 6a bottom).

It should be noted that, due to the high green autofluorescence signal of the bundles, the cell orientation was analyzed using the images of 20x magnification (Figure 6a). A color‐coded orientation map was generated from these images, where each hue represented a specific alignment angle. The image areas were quantified to evaluate myotube orientation within ±10° and ±20° ranges of a 180° distribution. Within ±20° deviations, 53.3% ± 14.34% of the detected myotubes cultured with fibrillar bundles exhibited a significantly greater alignment, compared to 35% ± 4.87% detected myotubes in the bioink printed without the bundles (Figure 6c). Observing the alignment of myotubes in all directions indicates that the greatest fraction (green arrow) of myotubes cultured with bundle fibers is growing between (−10°) to (+10°) (Figure 6b, right) and (−5°) to (+5°) (Figure S5a, Supporting Information, right). In contrast, the largest percentage (green arrow) of myotubes cultured without fibrillar bundles seemed to grow in the direction between (−10°) to (−30°) (Figure 6b left) and (−15°) to (−25°) (Figure S5a, Supporting Information left). Within ±10° deviations, the fraction of aligned myotubes in the composite bioink accounted for 29% ± 12.32%, which did not significantly differ from the bioink without the bundles, which showed growth of 20% ± 8.9% aligned myotubes (Figure S5a,b, Supporting Information). Notably, myotube alignment appeared more pronounced when myofibers were located closer to the bundles (Figure S5a, Supporting Information). This effect varied across different z‐planes, reflecting differences in the distance from the bundles.

Comparing the width of myotubes cultured without fibrillar bundles (51.6 ± 12.5), with the width of myotubes on the fibrillar bundles (28 ± 8.7), or next to the bundles (20 ± 4.2), revealed a significant difference (Figure S5d, Supporting Information). This is in accordance with Koeck et al.^[^ ^20^ ^]^ culturing myoblasts and differentiating them on wet‐spun collagen fibers. They reported a smaller width of differentiated myotubes grown on wet‐spun fibers and a higher aspect ratio (length to width) in comparison to the cells grown on a tissue culture plate. Also, Juhas et al.^[^ ^83^ ^]^ measured a much lower width of 8.73 ± 0.33 µm after one week and 22.2 ± 1.33 µm after four weeks of differentiation using pillar stretching stimulation. A higher myotube diameter of 13.5 ± 1.5 µm via stretching stimulation was reported in the work of Madden et al.^[^ ^84^ ^]^ Cells close to fibrillar bundles or on them will use the fibrillar topography with a diameter of a few hundred nanometers as contact guidance to grow more longitudinally, fuse, and form long myotubes. The fibrillar bundles also enlarge the surface area in the ink; therefore, they demand a higher cell load to reach the same myotube thickness as the control. This explains the appearance of a smaller diameter of myotubes measured for these cells in comparison to the cells in the bioink without fibrillar bundles. The results highlight the potential of PCL fibrillar bundles to promote myotube alignment, as a key requirement for the fabrication of the hierarchical architecture of skeletal muscle tissue, which can be further applied as a model for therapeutic screening, muscle‐related diseases, or as a potential tissue transplantation.

While various tissue engineering approaches have been developed to generate densely aligned myotubes and enhance directional contraction, quantitative assessment of alignment has often received less emphasis.^[^ ^15^, ^83^, ^85^, ^86^, ^87^ ^]^ For instance, Raman et al. reported the formation of densely aligned myotubes arranged in a rubber band–like ring structure around micropillars under mechanical stretching stimulation; however, quantitative alignment data were not reported. In contrast, Gao et al.^[^ ^12^ ^]^ and Ebrahimi et al.^[^ ^15^ ^]^ quantified a very high cell alignment of 91.37% and 90.1% respectively, but on a 2D level using a micromolding or patterning technique. Bian et al.^[^ ^88^ ^]^ adapted the micromolding approach for 3D constructs by embedding cells and fibrin gel into elastomeric molds containing staggered hexagonal posts, achieving an alignment of 80.0% ± 0.03%. However, while stretching‐based alignment strategies using posts or pillars may achieve high degrees of alignment, they are limited by the geometry size and dimensions of the pillars themselves. In contrast, bioprinting offers the possibility of fabricating tissue transplants customized to the patient's needs. This is particularly relevant since not all skeletal muscles exhibit a fusiform architecture shaped by pillar‐like structures; some, such as those in the hand or shoulder, display a pennate organization.^[^ ^89^ ^]^

The limitations of cell alignment via composite fiber bundles lie within the fiber packing density and the bioink development. Nonetheless, fiber incorporation in GelMA bioink is restricted, as densities above ≈3% reduce alignment, while even higher concentrations increase the risk of clogging due to entanglement of the bundles (Figure S2b, Supporting Information). Enhancing ink viscosity either by increasing hydrogel concentration or adding viscosity modifiers could improve fiber dispersion and facilitate improved fiber extrusion. However, current formulations capable of reaching such viscosities may compromise cell viability and proliferation through shear‐induced cell death, small pore size, excessive stiffness, and insufficient gel degradability caused by increased crosslinking density.^[^ ^33^, ^90^ ^]^ Further optimization is therefore required; once addressed, higher fiber concentrations and improved alignment may be achievable, ultimately promoting enhanced cellular organization. Alongside a viscous ink that still supports proliferation, other future improvements for our system could include fabricating thinner bundle fibers or further optimizing printing parameters such as deposition speed to enhance bundle alignment and promote consistent myotube orientation. Alternatively, other external stimulation, such as electric^[^ ^91^ ^]^ or magnetic fields,^[^ ^92^ ^]^ could guide the cell assembly with higher alignment and improve myogenesis and contractility.

## 3 Conclusion

In summary, we present fibrillar bundles made by the MEF technique as a promising bioink additive for achieving scalable, 3D cellular alignment in bioprinted tissue constructs, through flow‐induced alignment during extrusion bioprinting. The fibrillar bundles support cell adhesion and orientation across various cell types, including myoblasts, and significantly enhance myotube alignment within GelMA hydrogels. While challenges remain, such as improving fibrillar bundle dispersion, increasing bundle loading in the ink, and balancing ink viscosity and printability with both cell viability and fibrillar bundle alignment, this system shows strong potential for fabricating aligned, anisotropic tissues such as skeletal muscle.

## 4 Experimental Section

### Materials

The Dulbecco's phosphate‐buffered saline (DPBS) was purchased from Merck. GMP‐approved GelMa X‐Pure MW 90 kDa with 80% methylation degree was purchased from Rousselot. The reagents for the cell culture are as follows: Trypan blue reagent, Phalloidin‐iFluor 555 (Abcam), Hoechst 33258, bovine serum albumin (BSA), Fluoroshield mounting medium, DAPI 4′,6‐diamidino‐2‐phenylindole dihydrochloride, Lithium‐Phenyl‐2,4,6‐trimethylbenzoyl‐phosphinat (LAP), and high glucose Dulbecco's modified Eagle's medium (DMEM) for C2C12 and SCP‐1‐TERT were purchased from Merck. DMEM high glucose medium, for fibroblasts and PDL‐hTERT, Donkey anti‐mouse IgG (H+L), Alexa Fluor FITC, and Myosin 4 monoclonal antibody (MF20) were purchased from Thermo Fischer Scientific. Instead, Cell‐tracker green CMFDA was purchased from Invitrogen, Insulin‐transferrin‐selenium Solution (ITS), Fetal calf serum (FCS), horse serum (HS), penicillin−streptomycin (pen/strep), and GlutaMax were purchased from Gibco. 2‐(4‐(2‐Hydroxyethyl) ‐1‐piperazinyl)‐ethane sulfonic acid (HEPES), Triton‐X 100, paraformaldehyde solution, and BSA were purchased from Carl Roth. Trypsin/Ethylenediaminetetraacetic acid (EDTA) and fetal bovine serum (FBS) were purchased from BioSell. The PROFUSE‐S1 supplement was purchased from ProFuseTechnology. Murine C2C12 myoblast cells were purchased from ATCC.

PCL (Purac PC12) was purchased from Corbion Purac, PVAc (30 kDa) was purchased from Kremer Pigmente GmbH & Co. KG. Dichloromethane, Pluronic, and sodium Hydroxide (≥98%, pellets anhydrous) were purchased from Sigma–Aldrich

### Methods

### Methods—Blending of the Polymer System

The blend of two polymers was prepared by dissolving PCL and PVAc in a 30/70% (w/w) mixture in dichloromethane. The solution was cast and air‐dried to create a thin film. Subsequently, the film was loaded into the MEW printing syringe.

### Methods—Preparation of the MEW Collector Array

The different collector arrays for the collection of the fibrillar bundles were composed of a thin metal plate with rectangular holes of various spacing, resulting in three different grid structures. Before printing, the collectors were coated with a thin layer of poly(vinyl alcohol) to facilitate easier removal of the bundle fibers, similar to described by Lamberger et al.^[^ ^53^ ^]^

### Methods—MEW

The polymer blend as cast film was processed via an in‐house MEW machine, which has been described in detail previously.^[^ ^93^ ^]^ The polymer blend was processed at 190 °C at the nozzle temperature and 160 °C in the syringe area. A 3 kV voltage difference was applied over the 22 G needle, at a printing distance of 2.2 mm. The printing occurred with a collector speed of 1850 mm s^−1^ perpendicular to the rectangular slits in a temperature‐controlled room at 20 °C and a typical humidity of 40%. The printing patterns were generated using the vector drawing method as described by Lamberger et al.^[^ ^94^ ^]^

### Methods—Bundle Cutting

After printing onto the grids, the fibers were transferred to a laser cutter, Trotec Rayjet (maximum Power 55 W), and cut according to the grid spacing. The process parameters were a single cut with a speed of 6% and a power of 5%.

### Methods—Fiber Washing Process

The grid with the laser‐cut fibers was submerged in 70% ethanol. After soaking, the bundles were dislodged and centrifuged at 1500 rpm for 10 min. Subsequently, all but the solution covering the pellet was removed. The tube was refilled with fresh 70% ethanol, centrifuged, and the process was repeated a total of three times. After the last pellet formation, the ethanol was completely removed, and pure water was added to completely cover the pellet. Lastly, the bundles were frozen and lyophilized.

### Methods—Hydrogel Preparation

The GelMA ink was prepared by dissolving 150 mg of UV‐sterilized GelMA in 1.9 mL of DMEM media and 100 µL of a 1% (w/v) LAP solution at 50 °C for 10 min without stirring, resulting in a 7.5% (w/v) GelMA solution with a final LAP concentration of 0.1% w/v. Prior to the addition of bundle fibers to the hydrogel, they were hydrophilized by submerging the weighted pellet in a 10 mM NaOH solution for 30 min, followed by neutralization via 10x PBS. Afterward, the solution was mixed 1:1 volume with GelMA to gain the final printing concentration of 3.75% w/v GelMA, including 0.05% LAP for crosslinking with 3% (w/v) bundle fiber content. For crosslinking, the gel was exposed for 5 s to 60 mW cm^−2^ at 405 nm.

### Methods—Rheological Characterization of the Bioinks

The recovery test was performed by subjecting the inks to alternating high and low shear rates, starting with 0.1 s^−1^, followed by 1 s^−1^, then 0.1 s^−1^, 2 s^−1^, and finally returning to 0.1 s^−1^. This test is particularly useful for assessing the extent to which the viscosity recovers after exposure to elevated shear rates (1 and 2 s^−1^), returning to its initial value at 0.1 s^−1^. All tests were performed with the Discovery Hybrid Rheometer (TA Instruments), equipped with a 25 mm, sand surface plate geometry. Prior to loading, the rheometer plate was pre‐heated to the extrusion temperature of 23 or 25 °C, respectively, and samples were allowed to equilibrate for 60 s to ensure thermal adaptation.

### Methods—Printing of Bundle‐Loaded Hydrogel

The composite bioink was 3D printed with two different printers. First, the fiber alignment study with 20–30% (w/v) Pluronic was carried out using a BioX (Cellink Bioprinting AB, Gothenburg, Sweden) at 23 °C, whilst 3%, 4% and 5% GelMA were printed with an extrusion‐based 3D printer 3D Discovery (RegenHU, Villaz–Saint–Pierre, Switzerland), equipped with a heated regulating printhead (RegenHU) at 25 °C with a printing pressure of 0.2–0.38 bar. Both composite inks were loaded with 3 % bundles and extruded via an 18 G, conical nozzle, with a printing speed of 5–10 mm s^−1^ onto a glass slide using the print base protocol reported by Lamberger et al.^[^ ^67^ ^]^ 20 Images were taken per formulation using the stereomicroscope (M205 FCA, Leica, Germany, or Zeiss SteREO Discovery V.20). The orientation of ≈350 bundle fibers for GelMA and 120 bundle fibers for pluronic, per condition, was analyzed via ImageJ. The shear stress in the needle was calculated for GelMA as mentioned by Lopez Hernandez et al.^[^ ^95^ ^]^ The wall shear stress(τ) in the nozzle was calculated for GelMA using the formula based on the Hagen–Poiseuille law^[^ ^63^ ^]^ to assess the effect of the used printing condition on cell viability. The R stands for the Radius of the nozzle (m), the ΔP corresponds to the pressure applied during printing (Pa), and L stands for the length of the nozzle (m).

(1) $$\tau =\left(R\Delta P\right)/2L$$

### Methods—2D Cell Culture on Fibrillar Bundles

1. Cell culture: BJ‐TERT human fibroblasts were cultured in DMEM high‐glucose medium. Murine C2C12 myoblast cells were cultured in DMEM high glucose medium comprising 20 mM HEPES buffer and 4 mM GlutaMax. Human periodontal ligament progenitor cells, PDL‐hTERT, and human bone marrow‐derived mesenchymal stem cells SCP‐1‐TERT cells were kindly gifted by Denitsa Docheva^[^ ^96^, ^97^ ^]^ and cultured in DMEM high glucose medium. All media were supplemented with 10 % fetal calf serum and 50 U mL^−1^ penicillin‐streptomycin.
2. Cytoskeleton staining: Fibroblasts and stem cells were cultured at a density of 50 000 cells per 2 mg of fibril bundles on an agarose‐coated surface to inhibit the adhesion of the cells to the surface underneath. 2D control wells were cultured at 54000 cells cm^−2^. On days one and three, cytoskeleton staining was performed. Briefly, the cells were fixed for 30 min at room temperature (RT) using 4% (v/v) paraformaldehyde, followed by permeabilization in 0.1 % Tween 20 for 15 min. Cells were blocked in 2 % BSA in PBS for 30 min and stained for 60 min at RT using 1:400‐diluted Phalloidin‐iFluor 555 in 1% BSA/PBS. For nuclei staining, 10 µg mL^−1^ Hoechst 33258 was applied for 10 min at RT. Samples were then covered in Fluoroshield mounting medium. The interaction of cells with fibril bundles was imaged using confocal microscopy (TCS SP8, Leica, Wetzlar, Germany).
3. Myotube staining: After three days of culture of C2C12 cells on fibrillar bundles, the cells were washed with 1x PBS and transferred to differentiation media containing high‐glucose DMEM, 2% horse serum, 1% penicillin‐streptomycin, 1% ITS, and 20 mM HEPES. To accelerate differentiation from 7 to 4 days, 0.1% Profuse was added only on day one. For staining for fast and slow myosin heavy chain (MF20), cells were washed with PBS buffer, fixed with 4% v/v paraformaldehyde, and permeabilized with 0.1% (v/v) Triton for 15 and 5 min at RT, respectively. To reduce the background, the aldehyde groups were blocked with 5%w/v BSA solution in PBS at 37 °C for 30 min. Afterward, the samples were incubated with the fast and slow myosin 4 (MF20) antibody in a 1:500 dilution in PBS 0.1% BSA at 4 °C overnight. The next day, the sample was washed and placed in the incubator for 1 h with the secondary antibody Alexa Fluor 488‐conjugated donkey anti‐mouse antibody in a 500x dilution in PBS 0.1% BSA and 300 nM DAPI prior to imaging using the confocal laser scanning microscope (TCS SP8 HyD, Leica, Germany). Five z‐stack pictures were taken per replicate with 10x and 20x objectives, and the myotube length, width, and myotube alignment were calculated via ImageJ. The DAPI signal of the nuclei was used to analyze the myotube orientation on the bundle fibers via OrientationJ and following the procedure introduced by Narayanan et al.^[^ ^80^ ^]^ and Wang.^[^ ^82^ ^]^

### Methods—3D Bioprinting of C2C12 with Fibrillar Bundles

1. Preparation of C2C12 GELMA Ink: Preparation of GelMA composite bioink: A cell pellet containing 0.5 million mL^−1^ C2C12 cells was resuspended in 2 mL of warm 7.5% GelMA ink by pipetting up and down to ensure homogeneous cell distribution. The cell‐laden bioink was then diluted to 3.75% with 2 mL of 6% bundle fiber suspension and transferred into 5 mL syringes. To promote uniform distribution of cells and bundle fibers within the bioink, the syringes were rotated at RT for a partial physical gelation process. The partially gelated bioink was subsequently incubated at 4 °C for 5 min, followed by an incubation at 25 °C in a heated print head for an additional 5 min, prior to 3D printing. After printing, GelMA was crosslinked for 5 s at 405 nm, with 60 mW cm^−2^, before the addition of the culture medium to the printed strands.
2. Viability staining: Printed C2C12 cells with and without fibril bundles were stained for their viability using the live/dead assay. Ethidium homodimer‐1 and calcein AM were used to stain the dead and live cells in red and green, respectively. The dyes were dissolved in PBS, following the manufacturer's protocol, and cells were incubated in the solution for 30 min at RT prior to imaging. Nine images were taken using the fluorescence microscope (Leica DMI 3000B, Wetzlar, Germany), equipped with LAS X software, and analyzed via ImageJ. The cell viability was calculated via the following formula.
(2) $$Cellviability\left(\%\right)=\left(Viablecells/totalcells\right)\;*100$$
3. *Cell tracker staining*: Trypsinized C2C12 cells were resuspended in culture medium with 0.385 mg mL^−1^ cell tracker green for 20 min, according to the manufacturer's protocol. The cells were centrifuged, and the pellet was washed with PBS three times prior to addition to the GelMA ink and 3D bioprinting. The labeled cells mixed with GelMA were imaged after one and three days of culture via fluorescence microscope with 10x and 20x magnification using FITC channel and brightfield.

### Methods—Myogenesis Analysis on 3D Bioprinted Composite Bioink Containing Fiber Bundles

The C2C12 cells were mixed with GelMA and bundle fibers, printed, and cultured until differentiation as explained in “2D cell culture on fibrillar bundle”. For staining the sarcomeric units for their slow and fast myosin heavy chain MF20, cells were washed with PBS buffer, fixed with 3.7% v/v paraformaldehyde, and permeabilized with 0.3% v/v Triton for 15 and 5 min at RT, respectively. To reduce the background, the aldehyde groups were blocked with 5% (w/v) BSA solution in PBS at 37 °C for 30 min. Afterward, the samples were incubated overnight with the primary antibody, myosin 4 monoclonal primary antibody (MF20) in 1:500 dilution in 0.1% BSA at 4C° overnight. The next day, the sample was washed and incubated for 1 h with the secondary antibody Donkey anti‐Mouse antibody Alexa Fluor Plus 647 in a 1:500 dilution in PBS 0.1% BSA and 300 nM DAPI prior to imaging using the confocal laser scanning microscope (TCS SP8 HyD, Leica, Germany) with 10x and 20x magnification. The Orientation of myotubes was calculated by generating a color mask via the analysis tool of OrientationJ. Afterward, the Color mask was used to calculate the abundance of aligned myotubes via the Color Threshold tool. The data was divided into fractions of 5.5% and 11% respectively, which represent 10° and 20° of the 180° distribution. The alignment of ±5 ° and ±10° in direction from 90° was calculated to evaluate the percentage of myotubes growing in the printing direction.

### Methods—Statistics

A one‐way analysis of variance (ANOVA) was performed using the Turkey post hoc test and Levene's test to check for variance homogeneity for bundle alignment tests using different concentrations of Pluronic and GelMA. The ANOVA was conducted using Origin (OriginLab 2022), using at least nine biological replicas per group for all cell studies. A Student's *t*‐test was conducted to test for significant differences between Myoblasts printing with and without bundles. The significance level was set as *p*< 0.05 = ^*^, *p*< 0.01 = ^**^, and *p*< 0.001 = ^***^. All results are presented as means or medians with ± standard deviation (SD).

## Conflict of Interest

The authors declare no conflict of interest.

## Author Contributions

S.H., Z.L., and L.S. contributed equally to this work. S.H., Z.L. and L.S. performed the conceptualization and formal analysis and wrote the original draft. M.R. and G.L. visualized the work. S.S., G.L. and M.R. edited the manuscript and did the supervision. The Funding was acquired by S.S., G.L. and M.R., and all authors have read and given their consent to the final version of the manuscript.

## Supporting information



Supporting Information

## Data Availability

The data that support the findings of this study are available in the supplementary material of this article.
