# Supplementary material for: Fibrillar Bundles as Fibrous Filler Materials for Attaining Cell Anisotropy in Bioprinting
Source: Adv Healthc Mater. 2025 Nov 7;15(6):e03767. doi: 10.1002/adhm.202503767 (PMC12892021; doi:10.1002/adhm.202503767)
Supplement: Supplementary file 1 — Supporting Information [file ADHM-15-0-s001.docx]

**Supporting Information**

**Fibrillar bundles as fibrous filler materials for attaining cell anisotropy in bioprinting**

*Sven Heilig^a+^, Zan Lamberger^a+^, Lys Sprenger^b+^, Vivien Priebe^a^, Camilla Mussoni^a^, Denitsa Docheva^c^, Kristina Andelovic^a^, Jürgen Groll^a^, Sahar Salehi^b^, Gregor Lang^a^*, Matthias Ryma ^a^**


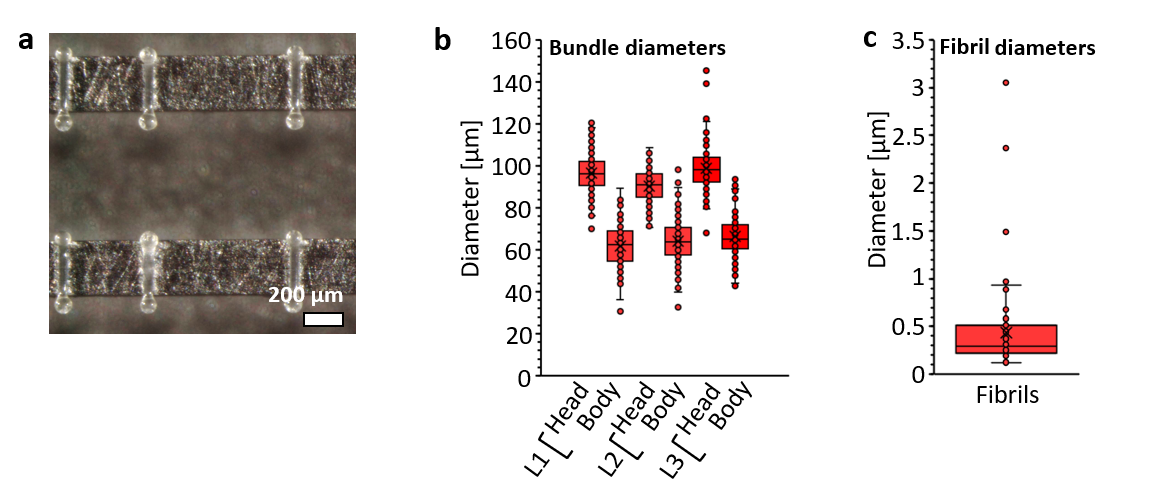


**Figure S1.** a) Fibers on the laser cutting grid after laser cutting. b) Diameter of the head and the body of the fibrillar bundles for the different bundle lengths. (n=190) c) Diameter of the fibrils composing the fibrillar bundles (n=80).


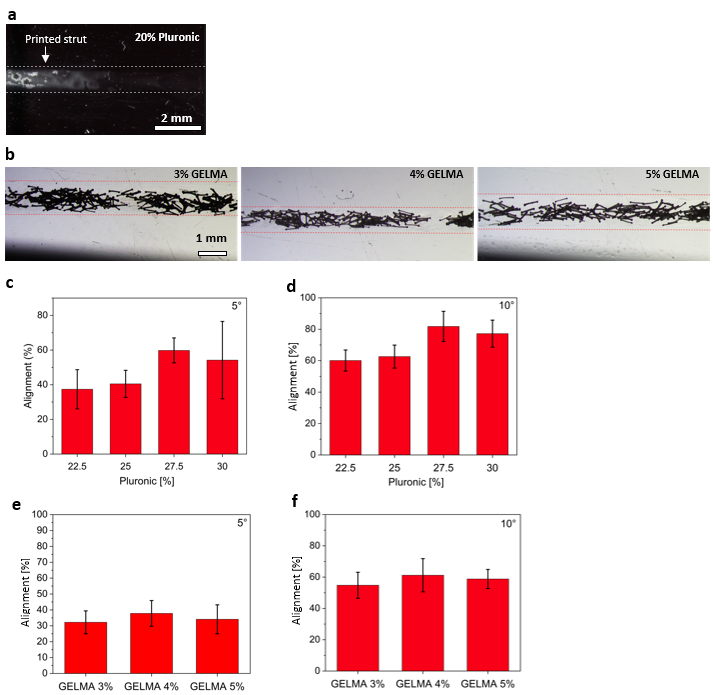


**Figure S2.** Printed composite ink containing 4% fibrillar bundles encapsulated in a) 20% (w/v) Pluronic, b) 3% (w/v) (left), 4% (w/v) (middle), and 5%(w/v) (right) GelMA, at 25°C, c) Alignment of 3% fibrillar bundles, encapsulated in 22.5, 25, 27.5 and 30% Pluronic within ±5° deviation from the printing direction, and d) ±10° deviation from the printing direction, e) Alignment of 3% fibrillar bundles encapsulated in 3, 4, and 5% GelMA within ±5° deviation from the printing direction and f) ±10° deviation from the printing direction.


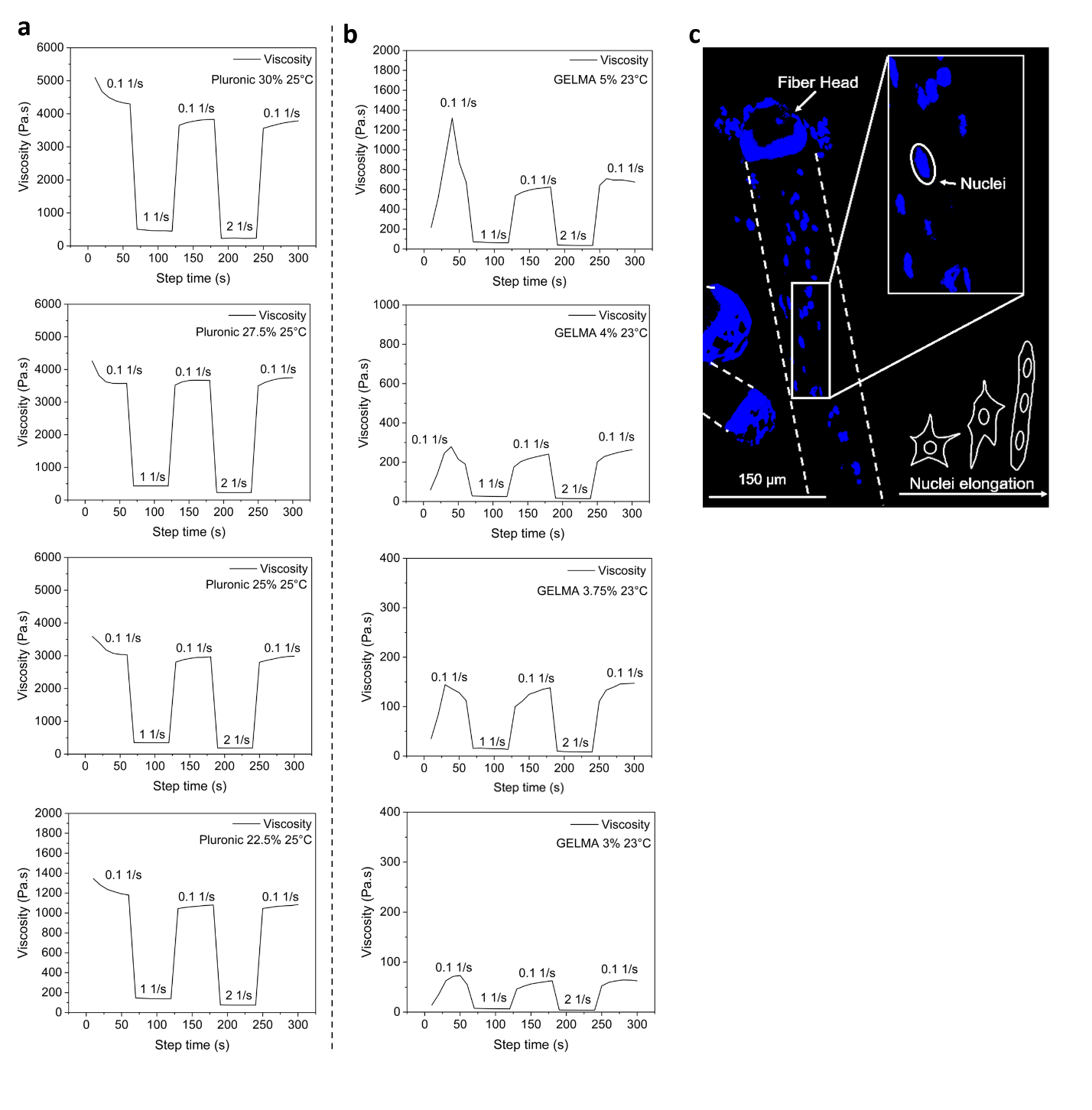


**Figure S3.** Viscosity diagrams for different concentrations of a) Pluronic and b) GelMA. c) Examplary image of the DAPI channel to show the elongated cell nuclei along the bundle fibers which is used for the evaluation in Figure 4d.

**
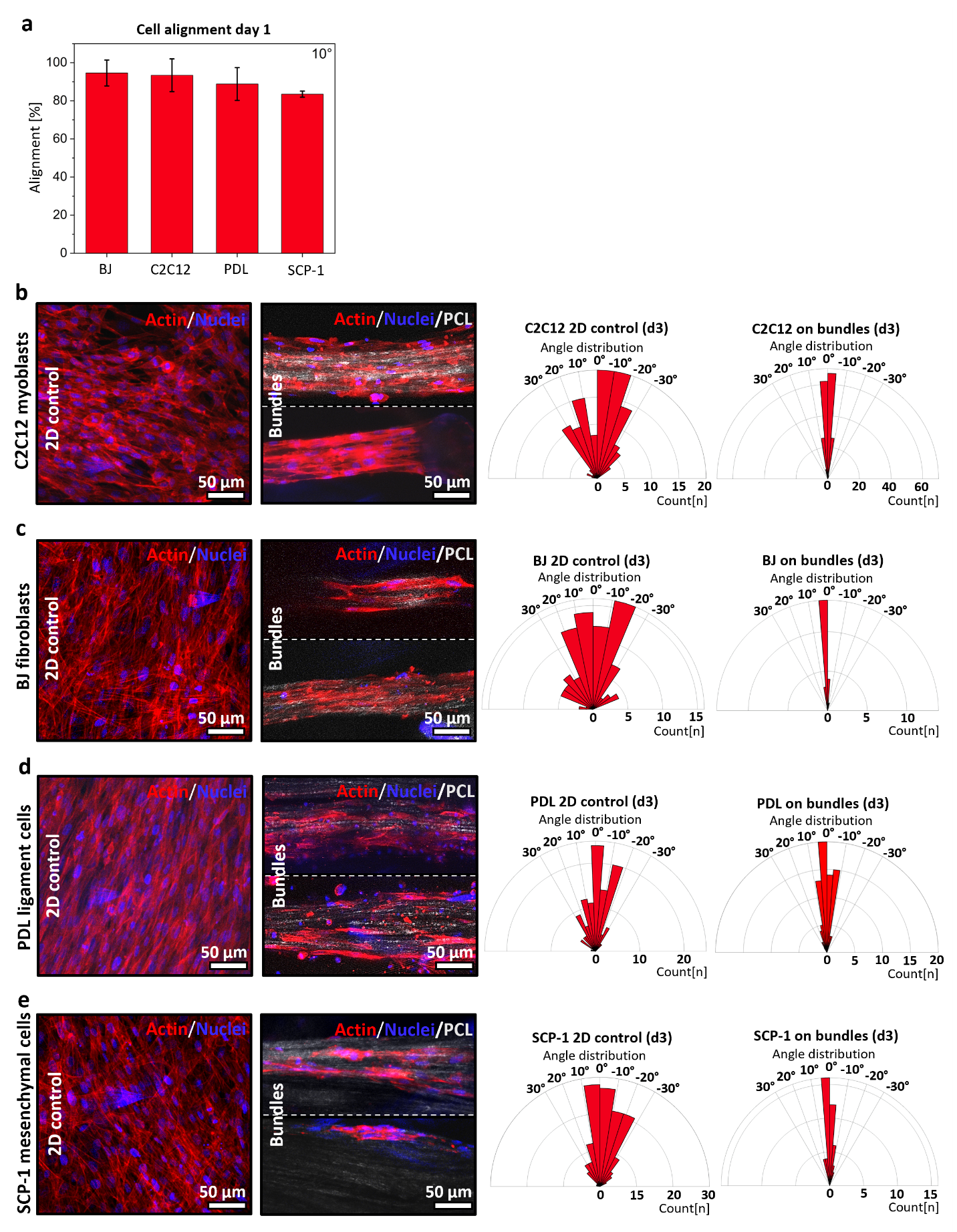
**

**Figure S4.** Cellular alignment of different cell lines imaged by confocal microscopy after staining the cytoskeleton actin filaments and nuclei using Phalloidin-iFluor 555 and Hoechst in red and blue, respectively. a) Cell alignment on day one along the bundles in 2D within a ±10° deviation from the fibrillar bundle direction. Cells on coated well plates are used as a control and compared with fibrillar bundles after three days of culture. The alignment of the cells based on a ±5° deviation of their cytoskeleton from the axis of the fragments was analysed and plotted for b) C2C12 murine myoblasts, c) BJ-TERT human fibroblasts, d) PDL-hTERT human immortalized periodontal ligament progenitor cell line, and e) SCP-1-TERT human mesenchymal bone marrow-derived stem cell line.


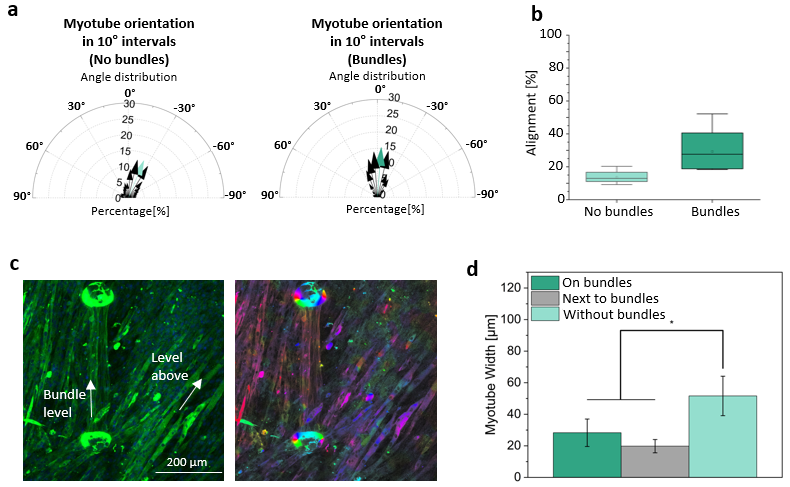


**Figure S5:** Myotube orientation analysis after 3D bioprinting of the bioink (3.75% GelMA and 3% fibrillar bundles) cultured for 3 days in growth medium and 4 days of differentiation, a) Compass plot of aligned myotube area (%) within ±10° deviation from the central 90° orientation, comparing printed constructs without (left) and with bundles (right). The green arrows indicate the predominant direction of myotube growth. b) Quantification of myotube orientation distribution in GelMA with and without bundles with ±10° deviation.
